# Reports on Hospitals of the United Kingdom

**Published:** 1914-07-25

**Authors:** Henry Burdett


### REPORTS ON

### Hospitals of the United Kingdom.

By SIR HENRY BURDETT, K.C.B., K.C.V.O.

SERIES III.

### THE ROYAL SOUTHERN HOSPITAL, LIVERPOOL.

We published an account, with plans, of the new out-patient department of this hospital on pages 415 to 417 of our issue of December 31, 1910. It has the great merit of being entirely distinct from the hospital proper, and, as our criticism showed, the site occupied is most appropriate, and possesses the great advantage of enabling the entrance for the patients to be arranged in one street with the exit in another, an arrangement which prevents overcrowding and the unnecessary intermingling of patients entering, awaiting treatment, or, on being treated, leaving the department. The plan of the hospital is pavilion, with straight blocks having two large wards on either side of the central or administrative ward building. The wards are airy and light, as well as being attractive in appearance, though they are much higher and contain more superficial and cubic space per bed than the latest approved plans now contain. The equipment of the wards is, on the whole, good. The equipment of the central blocks and offices connected with the wards displays the need of better fittings in many parts of the building, and points to the exercise of so-called economies in the wrong direction—that is in the omission to supply such things as an efficient safe for the food which it is found necessary to retain on each floor. There were some ice-safes, but the pattern was faulty, and in no case did we find these safes equipped and in order.

### Condition of Appliances and Storage.

This illustrates the right principle that there is nothing so fatal to efficiency in a hospital as the introduction of what are called cheap appliances, which in practice mean defective and often useless articles. It is far better to do without and to wait for the best rather than to temporise with cheap substitutes that can never prove satisfactory when put to the test of the hard wear necessitated by hospital use.

We were much struck by the number of wards where the baths were not in a condition to be used owing to being crowded with flower-pots and other things, pointing to the use of the bath for watering the plants and flowers—a practice which leads to the clogging of the pipes and other insanitary and objectionable things. There were two notable exceptions to this lack of a standard of the highest excellence in the administration of the wards. In one case the excellent efficiency of the ward was due to the spirit and zeal of the charge nurse, who was acting as sister in the absence on leave of that official; and the other case was that of two of the large wards under one sister who is evidently most efficient, most conscientious, and most admirable, not only in her own work, but in the discipline which she is able to enforce, and the good order and system which are strikingly noticeable in all the arrangements of the department of the hospital under her charge.

It is most unfortunate that the storage system for food, vegetables, meat, milk, and other matters is situated in the basement, which is dark and ill-ventilated, despite the fact of the wire doors and windows which have been introduced there. Liverpool is noted for the generosity of many of its citizens, and we could wish that some man or woman of intelligence and good-will could visit the stores department of this hospital, and leave a cheque with the secretary to defray the cost of supplying the hospital with proper larder and other accommodation situated above ground, and so planned as to give every portion of it the maximum of air and light. In the absence of these essentials, hygienic efficiency and the supply of the freshest and best foods in the finest condition are not obtainable.

### The Want of Income.

There is no doubt that this hospital is, we are afraid, necessarily starved for want of sufficient income. Were this not so the repairs and maintenance would receive more attention, and the whole place would be made bright by closer attention to decorative repair and the addition of many little things which add immensely to the comfort of the patients and to the smartness of the wards and their adjuncts.

On the top floor of one of the pavilions is situated a tropical diseases ward with two class-rooms or laboratories for the use of the students. The latter rooms would be all the better for the expenditure of a little more money upon their upkeep and regular cleansing and maintenance. There is an ample kitchen with all reasonable facilities for the proper and appetising preparation of food of all kinds. Several portions of the buildings which properly belong to the kitchen unit need a thorough overhaul and purification, but several of them can never be really efficient for their purpose until they are taken out from the cellars and put upon terra firma.

### The Work of the Hospital.

This hospital fulfils a very useful purpose by ministering to the needs of a large population of poor people who reside in its immediate vicinity. For this reason it is a hospital which ought to have the support of all classes of men and women in Liverpool who take a thoughtful and real interest in the welfare and care in sickness of their humbler neighbours. Every hospital subscriber might properly spare a small sum every year for the Royal Southern Hospital, whatever other hospitals they may support by preference or for other reasons.

The Tropical Wards.

The patients in the tropical wards—who, we were glad to hear, are most amenable—and also in the general and special wards throughout the building, were cheerful and evidently well satisfied with their treatment and surroundings. We noticed, however, in several instances that two children were put in the same bed in the children's ward, a state of affairs which, however great the pressure may be, ought never to be allowed to occur in a hospital of the size and importance of the Royal Southern Hospital, Liverpool. It must be a most onerous and difficult task to administer this hospital and keep it going throughout the year in the face of the fact that the funds at the disposal of its managers are never quite enough, and so far from ample, having regard to the amount of work done and the efforts made to secure economical administration in all departments. Our visit was made without notice on Sunday afternoon, June 21, and we consequently missed seeing the matron and some other officials with whom we should have been glad to have had an opportunity of discussing several points on their merits. Nearly 3,000 in-patients and upwards of 14,500 out-patients were admitted to treatment during 1913.

### THE HOLMESDALE COTTAGE HOSPITAL, SEVENOAKS.

This hospital stands high and has an attractive appearance. It has a nice garden, in which is the mortuary building, being situated at a good distance from the hospital. We visited it on the afternoon of November 4, 1913, and found the matron, Miss Siviour, an old Middlesex-trained nurse, energetically engaged and busily at work. We went through the whole institution with great pleasure because it provided abundant evidence of the excellent way in which the staff, consisting of the matron and a trained nurse and two probationers, carry on the work.

There were a small but efficient operation theatre, an abundance of linen and other cupboards, an excellent kitchen, ample lavatories, good baths and wards, the whole of which we found in good order and smartly administered.

The hospital has twelve beds, and up to the passing of the National Insurance Act the pressure on the beds was so great that a movement was started to build a new hospital as a memorial to King Edward VII. At the time of our visit the pressure had diminished considerably on the women's side, a fact attributed to the National Insurance Act. We think it is fortunate that no steps have been taken so far to reconstruct this attractive little hospital, much less to take a new site and to build an entirely new one.

Our view is, from the best knowledge we have been able to obtain, that the managers will be well advised not to engage in building operations of any kind for at least another year, so that they may be in a position to realise the probable effects of the changing conditions which all hospitals must prepare for in the near future. If such reconstruction and enlargement are decided upon, we hope they will include the proper ventilation of the mortuary and the addition to it of a chapel or view room. Then this unit should exhibit due reverence for the dead and full regard for the feelings of the friends.

